# The Effects of Acute and Chronic Sprint-Interval Training on Cytokine Responses Are Independent of Prior Caffeine Intake

**DOI:** 10.3389/fphys.2018.00671

**Published:** 2018-06-05

**Authors:** Guilherme A. Ferreira, Leandro C. Felippe, Rômulo Bertuzzi, David J. Bishop, Emiliano Barreto, Fernando R. De-Oliveira, Adriano E. Lima-Silva

**Affiliations:** ^1^Sports Science Research Group, Academic Center of Vitoria, Federal University of Pernambuco, Recife, Brazil; ^2^Endurance Performance Research Group, School of Physical Education and Sport, University of São Paulo, São Paulo, Brazil; ^3^Institute of Sport, Exercise and Active Living, Victoria University, Footscray, VIC, Australia; ^4^School of Medical and Health Sciences, Edith Cowan University, Joondalup, WA, Australia; ^5^Cell Biology Laboratory, Biological and Health Sciences, Federal University of Alagoas, Maceió, Brazil; ^6^Center for Studies of Human Movement, Department of Physical Education, Federal University of Lavras, Lavras, Brazil; ^7^Human Performance Research Group, Technological Federal University of Parana, Curitiba, Brazil

**Keywords:** exercise, interleukin-6, interleukin-10, tumor necrosis factor-alpha, over-reaching

## Abstract

We examined the effect of acute and chronic sprint interval training (SIT), with or without prior caffeine intake, on levels of exercise-induced inflammatory plasma cytokines [interleukin (IL)-6, IL-10, tumor necrosis factor (TNF)-α]. Twenty physically-active men ingested either a placebo (*n* = 10) or caffeine (*n* = 10) 1 h before each SIT session(13-s × 30-s sprint/15 s of rest) during six training sessions (2 weeks). The early (before, immediately after, and 45 min after the exercise) and late (24 and 48 h after the exercise) cytokine and creatine kinase (CK) responses were analyzed for the first and last training sessions. Plasma IL-6 and IL-10 peaked 45 min after the exercise, and then returned to basal values within 24 h (*p* < 0.05) in both groups on both occasions (*p* > 0.05). On both occasions, and for both groups, plasma TNF-α increased from rest to immediately after the exercise and then decreased at 45 min before reaching values at or below basal levels 48 h after the exercise (*p* < 0.05). Serum CK increased from rest to 24 and 48 h post-exercise in the first training session (*p* < 0.05), but did not alter in the last training session for the PLA group (*p* > 0.05). Serum CK was unchanged in both the first and last training sessions for the CAF group (*p* > 0.05). Two weeks of SIT induced a late decrease in the IL-6/IL-10 ratio (*p* < 0.05) regardless of caffeine intake, suggesting an improved overall inflammatory status after training. In conclusion, a single session of SIT induces muscle damage that seems to be mitigated by caffeine intake. Two weeks of SIT improves the late SIT-induced muscle damage and inflammatory status, which seems to be independent of caffeine intake.

## Introduction

A body of evidence suggests that sprint interval training (SIT) is a time-efficient mode of training that is able to promote central and peripheral adaptations associated with aerobic fitness (Gibala et al., 2006; MacInnis and Gibala, 2017). A typical SIT session is characterized by intermittent periods of effort performed at different exercise intensities, including self-paced, ‘all-out’ efforts, separated by periods of recovery (MacInnis and Gibala, 2017). The greater effort evoked during SIT stimulates a transient metabolic disturbance (i.e., greater 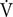
O_2_ and lactate accumulation), which will promote the activation of signaling proteins necessary for training-induced adaptation (Gibala et al., 2009; MacInnis and Gibala, 2017). Also, a SIT session promotes a transient, rich milieu of pro- and anti-inflammatory signaling cytokines, such as interleukins (IL)-6 and IL-10, and tumor necrosis factor (TNF)-α (Richardson et al., 2016), which may induce immune system adaptations to SIT.

However, the chronic effect of SIT on anti-inflammatory status has received limited attention. The few existing studies have reported conflicting results regarding the effects of a brief period of SIT on the exercise-induced inflammatory profile (Hovanloo et al., 2013; Richardson et al., 2016). In those studies, 2 weeks of SIT (4–7 × 30-s sprint/240-s rest) either did not affect the late (48 h) post-exercise IL-6 and IL-10 response (Hovanloo et al., 2013) or promoted a greater post-exercise increase in both IL-6 and TNF-α (Richardson et al., 2016). This is interesting because a late post-exercise increase in IL-6 and TNF-α, and an imbalance between pro- and anti-inflammatory cytokines (i.e., a greater IL-6/IL-10 ratio), may lead to an amplified inflammatory response that could result in immune suppression and chronic fatigue (Smith, 2000; Bessa et al., 2016), while a late reduction in post-exercise IL-6 and TNF-α and in the IL-6/IL-10 ratio might indicate a positive training adaptation of the immune system (Smith, 2000; Bessa et al., 2016). On the other hand, when IL-6 is acutely produced by exercising muscles (measured immediately after exercise), it could have anti- rather than pro-inflammatory proprieties as IL-6 will stimulate anti-inflammatory cytokines such as IL-10 (Starkie et al., 2003; Petersen and Pedersen, 2005). In addition, the acute release of IL-6 is also a marker of muscle metabolism (Carey et al., 2006; Pedersen and Febbraio, 2008; Pedersen, 2012). In this regard, interventions to enhance the acute post-exercise response of IL-6, and consequently IL-10, and to mitigate the late post-exercise imbalance in the IL-6/IL-10 ratio, might represent an interesting approach to attenuate the onset of harmful inflammatory processes (Kaspar et al., 2016).

A potential agent able to influence the overall inflammation status, which might act synergistically with SIT, is caffeine (Tauler et al., 2016). Previous studies have demonstrated that caffeine intake increases both plasma IL-6 and IL-10 immediately after a 15-km running competition when compared to a placebo (Tauler et al., 2013). Because a higher acute release of IL-10 might attenuate the late post-exercise production of both IL-6 and TNF-α (Steensberg et al., 2002, 2003), it could be argued that caffeine may have a late anti-inflammatory effect. Furthermore, studies using animal models have demonstrated that continuous exercise combined with caffeine has an anti-inflammatory effect (Cechella et al., 2014). However, the effect of caffeine on the exercise-induced increase in cytokines has only been investigated following a single session of continuous exercise, and its effects on SIT-induced adaptations of the cytokine response are unknown.

Therefore, this study was undertaken to investigate the effect of acute (one session) and chronic (2 weeks) SIT on early and late cytokine responses, and whether caffeine intake before training sessions may change these responses. We hypothesized that 2 weeks of SIT would change the post-exercise response to a more anti-inflammatory profile, and that pre-exercise caffeine intake would lead to a larger improvement in the anti-inflammatory environment after training.

## Materials and Methods

### Participants

Prior to joining the study, participants had their physical activity level analyzed by completing a short-version of the International Physical Activity Questionnaire. All participants were classified as physically-active, and their main characteristics are reported in **Table 1**. Participants were not consumers of dietary supplements such as taurine, branched chain amino acids, creatine, or any other supplement that could influence the main outcomes of the present study. A questionnaire was used to establish regular daily caffeine intake (Landrum, 1992). Mean habitual caffeine consumption was estimated to be 100 ± 72 mg day^-1^. The intake frequency of tea, cocoa, chocolate, and soda was less than once a week and in a small portion (∼240 mL week^-1^).

**Table 1:** Main characteristics of the participants and physiological parameters identified during an incremental test pre- and post-training.

	Placebo (*n* = 10)		Caffeine (*n* = 10)	
	Pre-training	Post-training	Pre-training	Post-training
Age (years)	25.2 ± 5.7	–	26.2 ± 4.5	–
Height (cm)	175 ± 11.0	–	178 ± 6.0	–
HCC (mg day^-1^)	118 ± 82	–	86 ± 68	–
Body mass (kg)	73.2 ± 10.2	73.4 ± 10.7	78.3 ± 8.9	78.3 ± 9.1
GET (mL kg^-1^ min^-1^)	26.0 ± 3.7	24.0 ± 3.0	24.5 ± 3.6	23.0 ± 3.3
GET (bpm)	140 ± 12	144 ± 19	147 ± 16	145 ± 18
GET (W)	123 ± 32	127 ± 20	125 ± 29	120 ± 20
RCP (mL kg^-1^ min^-1^)	31.0 ± 4.7	32.4 ± 2.4	30.3 ± 3.3	29.9 ± 3.9
RCP (bpm)	162 ± 15	166 ± 16	163 ± 13	168 ± 17
RCP (W)	171 ± 32	180 ± 32	161 ± 34	169 ± 35
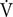 O_2max_ (mL kg^-1^ min^-1^)	37.9 ± 5.8	39.4 ± 4.8*	36.0 ± 5.5	38.9 ± 6.6*
HR_max_ (bpm)	184 ± 12.0	184 ± 13	186 ± 150	188 ± 15
Ẇ_max_ (W)	219 ± 36.0	227 ± 36*	214 ± 45	225 ± 34*

*HCC, habitual caffeine consumption; GET, gas exchange threshold; RCP, respiratory compensation point; 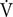
*O*_*2max*_, maximal oxygen uptake; HR_*max*_, maximal heart rate; Ẇ_*max*_, maximal aerobic power. ^∗^Significantly different from pre-training, *p* < 0.05. Data are mean ± standard deviation*.

This study was carried out in accordance with the recommendations of the Brazilian Research Resolution 466/2012. The protocol was approved by the Ethics Committee for Human Research at the Federal University of Pernambuco. All subjects gave written informed consent in accordance with the Declaration of Helsinki.

### Experimental Design

**FIGURE 1:**
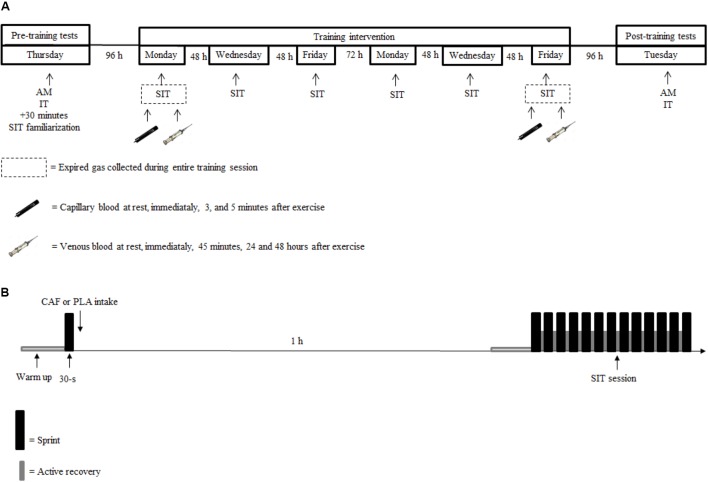
Experimental design of the study **(A)**, sprint interval training (SIT) session **(B)**. AM, anthropometric measurements; IT, incremental test; SIT, sprint interval training session; 30-s, baseline 30-s *all-out* sprint.

This longitudinal, placebo-controlled, and double-blind study was divided into three phases: (1) pre-training tests, (2) training intervention, and (3) post-training tests (**Figure 1**). Participants were instructed to refrain from exercise, alcohol, or any supplementation during the entire study. This began 48 h before the first test session and was maintained until the last test session. Participants were asked not to consume any food or drink that contained caffeine 24 h before each training session and during the 48 h after the first and last training sessions. They were given a list with all foods and drinks that contain caffeine and adherence to the recommendation was checked via a food diary. However, a cup of coffee (45 mg of caffeine) was allowed at breakfast on the days with no training. Participants also registered all foods and beverages ingested during the 24 h before the first test session, and replicated this diet during the 24 h before subsequent tests and training sessions.

### Pre-training Test

During the pre-training test session, participants filled out a 24-h food diary and underwent anthropometric measurements consisting of height and mass. All participants then completed an incremental test to exhaustion to determine their gas exchange threshold (GET), respiratory compensation point (RCP), maximal oxygen consumption (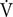
O_2max_), maximal aerobic power (Ẇ_max_), and maximal heart rate (HR_max_).

The incremental test was performed on a standard bicycle attached to an electromagnetically-braked roller (CompuTrainer Lab, RacerMate, Seattle, WA, United States), which was calibrated before each test as previously described (Davison et al., 2009). The optimal seat and handle position was recorded and reproduced throughout the study for each participant, to ensure an identical body position in all training and test sessions. Participants warmed up at 70 W for 5 min and thereafter power output was increased by 30 W every 3 min until exhaustion. Participants were instructed to maintain a pedal cadence of between 70 and 80 revolutions per minute (rpm). Exhaustion was defined as an inability to maintain a pedal cadence above 70 rpm. Oxygen uptake (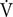
O_2_), carbon dioxide production (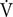
CO_2_), and ventilation (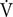
_E_) were measured breath-by-breath using an automatic metabolic cart (Cortex Metalizer II, Cortex Biophysik GmbH. Leipzig, Germany), and then averaged every 30 s. The metabolic cart was calibrated before each test with a 3-L syringe and a standard gas of established O_2_ and CO_2_ concentrations (12 and 5%, respectively). Heart rate (HR) was measured using a heart monitor transmitter (Polar, Kempele, Finland) connected to the metabolic cart (Cortex Metalizer). The GET was identified by three independent investigators as the exercise intensity corresponding to the lowest 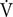
_E_/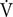
O_2_ ratio (Von Duvillard et al., 1993). Similarly, the RCP was identified by three independent investigators as the exercise intensity corresponding the point of a non-linear increase in the 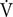
_E_/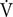
CO_2_, a constant increase in the 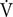
_E_/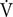
O_2_, and the first decrease in the expiratory fraction of CO_2_ (Meyer et al., 2005). The 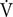
O_2max_ and HR_max_ were determined as the highest 30-s value recorded during the last stage of the test. The Ẇ_max_ was considered the highest power completed for 3 min during the test. When the last stage was incomplete (i.e., less than 3 min), the Ẇ_max_ was determined as the power of the last complete stage plus the product of the fractional time of the last incomplete stage by the incremental rate.

Thirty minutes after the incremental test participants performed a short SIT protocol (six sprints only) on the same bicycle attached to the electromagnetically-braked roller (CompuTrainer Lab, RacerMate, Seattle, WA, United States) to familiarize themselves with the SIT procedures.

### Training Intervention

Participants were divided using a matched-pairs design (Thomas et al., 2015). They were first paired based on their GET, RCP, 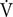
O_2max_, and Ẇ_max_. Then, individuals of a given pair were randomly assigned to one of two groups: placebo (PLA) or caffeine (CAF). This procedure guaranteed that participants of both groups had a similar initial level of physical fitness (**Table 1**). Participants performed six training sessions over a 2-week period. Training sessions were performed on Monday, Wednesday, and Friday and consisted of 13-s × 30-s *all-out* sprints interspersed by 15 s of active recovery. The shortest recovery (15 s) was used to prevent a reduction in 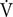
O_2_ during recovery, which allowed participants to cycle close to their 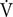
O_2max_ throughout each training session (Ronnestad et al., 2015). The first training session was initiated 96 h after the pre-training procedures. Tests and training sessions were always performed between 17:00 and 18:00 h to minimize the effects of circadian rhythms on sprint performance and circulating cytokines (Zhou et al., 2010).

On the training day, all participants arrived at the laboratory after a 2-h fast and rested for 20 min sitting on a chair. Thereafter, participants warmed up at 90% of their GET for 3 min, and then performed a baseline 30-s *all-out* sprint. This baseline sprint was used to determine training-induced adaptations in the ability to perform a single sprint without the confounding influence of caffeine intake. Thereafter, participants ingested either caffeine or a placebo and rested for 1 h until starting the SIT. Participants in the CAF group ingested a capsule containing 5 mg kg^-1^ of caffeine (Pharmapele Pharmaceutical Company S/A, PE, Brazil) 1 h before each training session, while participants in the PLA group ingested a capsule containing 5 mg kg^-1^ of cellulose (Pharmapele Pharmaceutical Company S/A, PE, Brazil) (Azevedo et al., 2016). Participants in the CAF group were classified as responders if their mean power output during the first sprint of the first training session increased at least 1% when compared with the mean power output at the corresponding baseline sprint. Six participants were considered responders.

The SIT session was initiated with a 3-min warm-up at 90% of the GET followed by 13-s × 30-s of *all-out* sprints, each separated 15 s of active recovery. Participants were encouraged to perform each sprint as fast as possible and to maintain a comfortable, self-paced cadence during the recovery periods. During the sprints, the cycle ergometer was set in pedal-rate-dependent mode and braking resistance kept constant. The braking resistance was kept constant through the RacerMaterOne software (CompuTrainer Lab, RaceMate, Seattle, WA, United States). CompuTrainer Lab is a load generator via electromagnetic eddy-current brake that fixes a given magnetic resistance on-the-fly based on bicycle weight (9 kg) and participant body mass. The gear ratio was fixed at 52 × 12 and participants were not allowed to alter this throughout the sprints. Therefore, power output was a function of the cadence. The power output was recorded second-by-second throughout each SIT session by the RacerMaterOne software.

During the first and last SIT sessions, gas exchange was continually monitored during the exercise (Cortex Metalizer II, Cortex Biophysik GmbH., Leipzig, Germany), and capillary blood samples were collected from the earlobe (40 μL) at rest, and immediately, 3 and 5 min after the exercise. The blood samples were immediately transferred to tubes containing 8 μL of EDTA, centrifuged for 10 min at 1000 × *g* at 4°C for plasma separation, and immediately analyzed for plasma lactate concentration ([La]). Venous blood samples (9 mL) were also taken at rest, and immediately, 45 min, 24 and 48 h after the first and last SIT session and collected in tubes containing separator gel (5 mL) or EDTA (4 mL). The samples were centrifuged within 30 min for 10 min at 1000 × *g* at 4°C (Mikro 220 centrifuge Andreas Hettich GmbH & Co. KG, Germany). The resulting serum/plasma was immediately stored at -20°C in 1 mL aliquots for subsequent analysis of serum creatine kinase (CK) concentration, or at -80°C in 1 mL aliquots for quantification of plasma cytokine levels.

The participants filled out a 24-h food diary before two randomly-chosen training sessions to check if they were maintaining the recommended diet. Food diaries were analyzed using Dietbox software (Mixpanel Mobile Analyst, San Francisco, CA, United States).

### Post-training Tests

Ninety-six hours after the last training session, participants performed the same tests as pre-training to determine physiological and morphological SIT adaptations.

### Analysis

Mean power (MP) was determined for each sprint. The MP was calculated as the mean power data from the 2^nd^ to the 29^th^ seconds of each sprint. The first and last seconds were excluded to avoid any noise related to reaction time at the beginning and end of the exercise. The 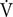
O_2_, 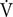
CO_2_ and 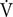
_E_ values were averaged for each sprint. The mean power output and corresponding mean 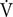
O_2_, 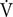
CO_2_ and 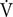
_E_ values during each 15-s recovery period were also calculated.

Plasma lactate and serum CK concentrations were determined by an enzymatic method using commercial kits (Labtest Diagnostica S.A., Minas Gerais, Brazil), with the resultant reaction read in a spectrophotometer (Genesys 10 S UV-vis, Thermo Electron Scientific Instruments, Madison, WI, United States). The 95% CI of the intra-assay coefficients for variation was 6.4–8.7%. Cytokines (IL-6, IL-10, and TNF-α) present in the plasma were quantified with a customized Milliplex Map Kit using a Magnetic Bead Panel I, 3-plex using a Luminex MAGPIX instrument and xPONENT MAGIPIX software (Luminex Corporation, Austin, TX, United States). The analysis was performed according to the manufacturer’s guidelines. The 95% CI of the intra-assay coefficients for variation in IL-6, IL-10, and TNF-α were 5.1–6.8, 5.4–7.0, and 8.9–12.8%, respectively. The IL-6/IL-10 ratio was calculated to represent the overall inflammatory status.

### Statistical Analysis

The required sample size was estimated using the G^∗^Power Package Statistical Power Analysis (Heinrich-Heine-University, Düsseldorf, Germany), using an effect size of 1.3 for the caffeine effect on the exercise-induced response of IL-6 (Rossi et al., 2017). Assuming an alpha error of 0.05 and a beta error of 0.80, the required sample size was estimated to be at least six participants in each group. The sample size was increased to 10 participants in each group assuming that some participants might drop out during the data collection. The Shapiro–Wilk test was used for check normality in the data distribution. Data for pre-training caffeine intake, IL-6, IL-10, TNF-α, and CK were non-normally distributed and were log-transformed before analysis. Due to technical problems with venous blood collection and the cytokine analysis, some time points were missed and the final sample size (*n*) is reported for all results. Macronutrient intake was compared using a two-way mixed linear general model with the group as the between factor and moment as the within factor. The MP during baseline sprints were compared using a two-way mixed linear general model with group as the between factor and training session as the within factor. Data for 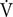
O_2_, and MP during SIT were compared using a three-way, mixed linear general model, with group as the between, and sprints (sprint 1 to sprint 13) and training session (first and last training sessions) as the within factors. A three-way, mixed linear general model, with group as the between factor, and acute response (rest, immediately, 45 min, 24 and 48 h after SIT) and training session (first and last training sessions) as the within factors was used to compare plasma IL-6, IL-10, TNF-α, and CK. Data for 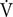
O_2max_, Ẇ_max_, HR_max_ and body mass were compared between groups and pre- and post-training intervention using a two-way, mixed linear general model. Fisher LSD *post hoc* tests were used to locate any differences between conditions and time points. Data normally distributed are presented as mean ± standard deviation (SD), unless otherwise stated. The level of significance was set at *p* < 0.05. All statistical procedures were performed in Statistic software version 10 (StataSoft, Inc.^®^, Tulsa, OK, United States).

## Results

### Food Records and Blinding

During the intervention period, as shown in **Table 2**, the values for total energy intake or macronutrients consumed for the PLA and CAF groups were not significantly different (*p* > 0.05). The protein intake and sources of protein (mainly meat and milk) were very similar between the groups. In addition, the protein intake (both as a percentage of total energy intake and relative to body mass) was maintained during the entire study without differences between the groups (*p* > 0.05). On average, the diet was composed of 43 ± 9% carbohydrate, 20 ± 5% protein, and 37 ± 7% fats for the PLA group and 46 ± 9% carbohydrate, 23 ± 7% protein, and 31 ± 4% fats for the CAF group. Habitual caffeine consumption was similar between groups (*p* > 0.05). The food record that participants completed during the training phase confirms that they followed the recommendations and did not ingest caffeine during this period. There was also no indication of caffeine intake over the 48 h after the first and last training sessions.

**Table 2:** Food records 24 h before (pre-training), and at two randomly-chosen training moments (moment 1 and 2), during the training intervention.

	Placebo (*n = 10*)			Caffeine (*n = 10*)		
	Pre-training	Moment 1	Moment 2	Pre-training	Moment 1	Moment 2
Carbohydrate (%)	45 ± 9	45 ± 8	40 ± 10	47 ± 10	46 ± 9	45 ± 7
Protein (%)	20 ± 6	19 ± 4	20 ± 6	22 ± 8	24 ± 7	23 ± 7
Lipid (%)	35 ± 7	36 ± 9	40 ± 6	31 ± 4	30 ± 5	32 ± 5
Total energy intake (kcal day^-1^)	2,503 ± 467	2,421 ± 738	2,429 ± 601	2,250 ± 584	2,184 ± 690	2,139 ± 651
Protein (g kg^-1^)	1.9 ± 0.6	1.6 ± 0.5	1.8 ± 0.8	1.6 ± 1.0	1.7 ± 1.0	1.8 ± 1.0
Caffeine intake (mg day^-1^)	118 ± 82	0 ± 0	0 ± 0	86 ± 68	0 ± 0	0 ± 0

*There was no difference between groups or moments, *p* > 0.05. Values are mean ± standard deviation*.

After the training intervention, only two participants (12.5%) in the PLA group correctly reported that they ingested the placebo. Only one participant (6.3%) in the PLA group thought he had ingested caffeine; the remaining (81.2%) reported they did not know what they had ingested. Only one participant (6.3%) in the CAF group reported correctly that he had ingested caffeine; the remaining (93.7%) said they did not know what they had ingested. There were no reported side effects related to caffeine intake.

### Sprint Performance and Physiological Responses During the First and Last Training Sessions

All participants were able to complete 13 sprints during the first training session. The MP during the baseline sprint before the supplementation increased from the first to last training session in both the PLA and CAF group (first training session, 5.6 ± 0.7; last training session, 6.0 ± 1.0 W kg^-1^, main effect for training session, *F*_(1,18)_ = 5.8, *p* = 0.03). There was no training and group interaction (*F*_(1,18)_ = 0.45, *p* = 0.51).

**FIGURE 2:**
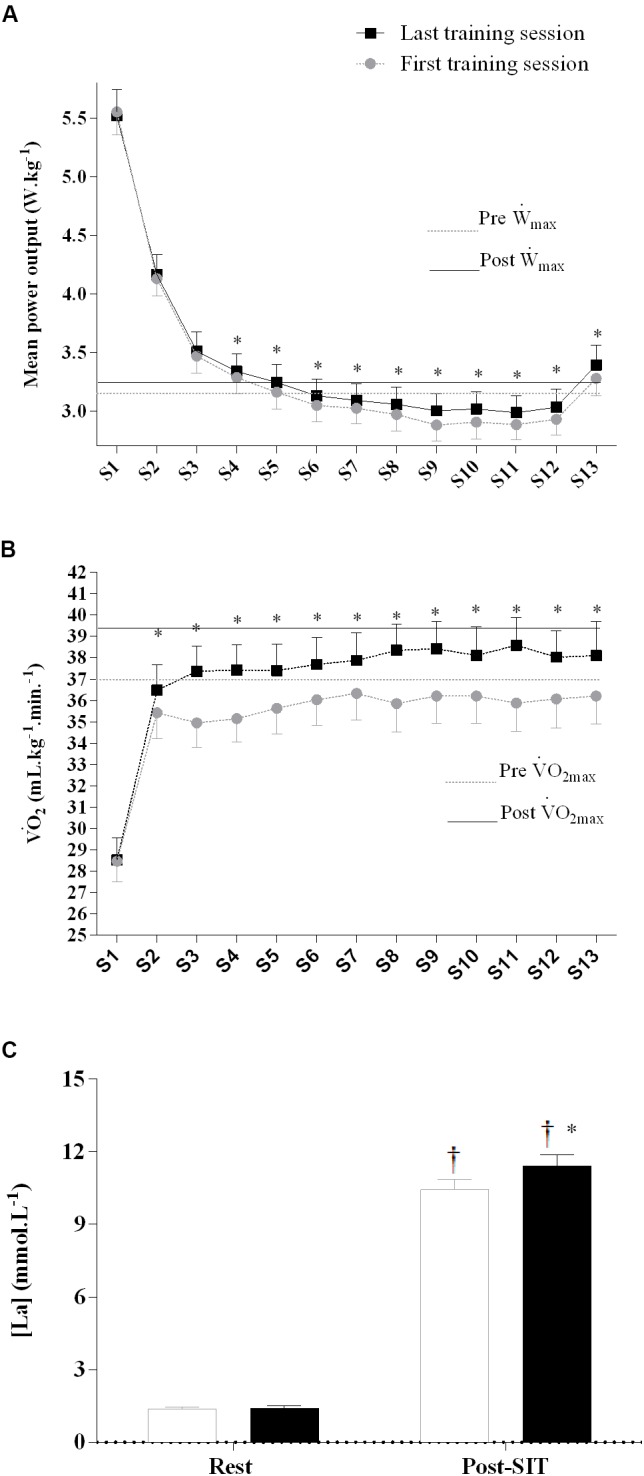
Mean power **(A)**, 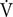
O_2_, oxygen uptake **(B)**, and resting and peak plasma lactate **(C)** response during the first and last training session. Placebo and caffeine groups have been pooled as there was no supplementation effect. ^∗^Significantly different from first training session, *p* < 0.05. ^†^Significantly different from rest, *p* < 0.05. Sprint effect (*p* < 0.05) is omitted for clarity (see text for details). Horizontal lines represent maximal values obtained in the incremental test pre- and post-training. Data are mean ± standard error of mean (*n* = 10 per group). S1–13, sprint 1 to 13.

The MP during sprints 4 to 13 was higher in the last than in the first training session (interaction between training session and sprint, *F*_(1,216)_ = 2.4, *p* = 0.01, **Figure 2A**), with no difference between the PLA and CAF groups (*F*_(1,18)_ = 2.1, *p* = 0.17). The 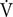
O_2_ was higher in the last than in the first training session from sprint 2 to sprint 13 (interaction between training session and sprint, *F*_(1,204)_ = 2.0, *p* = 0.02, **Figure 2B**), without differences between the PLA and CAF groups (*p* > 0.05). There was an interaction between time and training session for plasma lactate (*F*_(1,17)_ = 7.3, *p* = 0.02, **Figure 2C**), with values post-SIT increasing more in the last than in the first training session, without differences between the PLA and CAF groups.

There was no effect of caffeine or training on MP during the 15-s recovery intervals (*p* > 0.05). However, mean 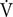
O_2_ during the work intervals was slightly higher in the last (37.0 ± 4.2 mL kg^-1^ min^-1^) than in the first training session (35 ± 5 mL kg^-1^ min^-1^) in both PLA and CAF (*p* > 0.05).

### Creatine Kinase Response During the First and the Last Training Sessions

**FIGURE 3:**
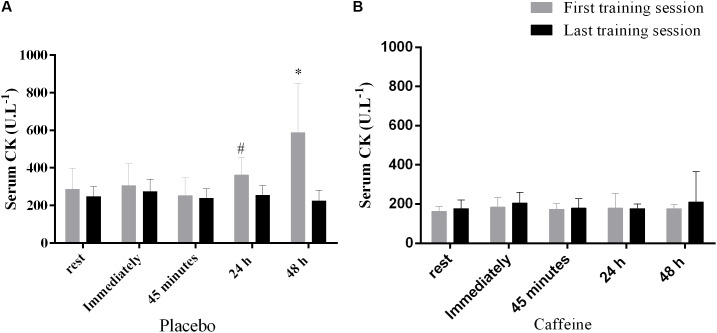
Serum creatine kinase after the first and last training session in the placebo **(A)** and caffeine **(B)** group. ^#^Significantly higher than at 45 min in the session 1, *p* < 0.05. ^∗^Significantly higher than at rest, immediately, 45 min and 24 h in the first training session, *p* < 0.05. Data are mean and standard error of mean (*n* = 6 and 7 in PLA and CAF groups, respectively).

The CK concentration increased from rest to 24 h and from 24 to 48 h after the exercise only after the first training session and only in the PLA group, but remained unaltered in the CAF group in both training sessions (Interaction between group, training session and time, *F*_(4,44)_ = 2.6, *p* = 0.049, **Figures 3A,B**).

### Cytokines Response During the First and the Last Training Sessions

**FIGURE 4:**
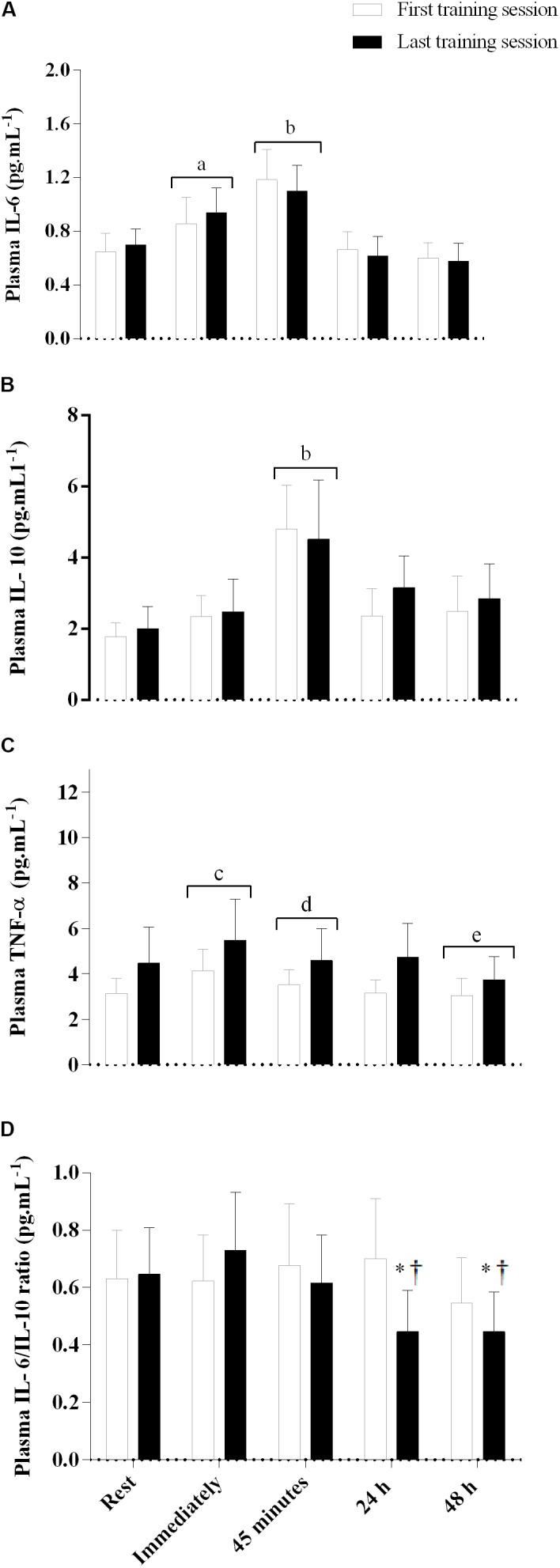
Plasma interleukin-6 **(A)**, interleukin-10 **(B)**, tumor necrosis factor (TNF)-α **(C)**, and IL-6/IL-10 ratio **(D)** immediately, 45 min, and 24 and 48 h after the first and last training sessions. Placebo and caffeine groups have been pooled as there was no supplementation effect. ^a^Significantly different from rest, 24 and 48 h after exercise; *p* < 0.05. ^b^Significantly different from rest, immediately, 24 and 48 h after exercise; *p* < 0.05. ^c^Significantly different from rest, 45 min, 24 and 48 h after exercise; *p* < 0.05. dSignificantly different from immediately and 48 h after exercise; *p* < 0.05. eSignificantly different from rest, immediately, 45 min, and 24 h after exercise; *p* < 0.05. ^†^Significantly lower than rest, immediately, and 45 min after SIT in the last training session; *p* < 0.05. ^*^Significantly lower than all time points in the first training session; *p* < 0.05. Data are mean and standard error of mean (*n* = 6 for PLA and 7 for CAF groups for IL-6 and IL-10, and *n* = 6 and 9 for TNF-α, respectively).

Plasma levels of IL-6 were elevated immediately after exercise, and increased further 45 min post-exercise; however, after 24 h the plasma levels of IL-6 returned to values equivalent to rest and remained at this level until 48 h post-exercise (main effect of time, *F*_(4,44)_ = 22.8, *p* = 0.001, **Figure 4A**). The plasma levels of IL-10 remained unaltered from rest to immediately post-exercise, but increased 45 min after the exercise, and then returned to basal values 24 h after the exercise (main effect of time*, F*_(4,44)_ = 11.5, *p* = 0.001, **Figure 4B**). The plasma levels of TNF-α increased from rest to immediately after the exercise, and decreased below basal values 48 h after exercise (main effect of time*, F*_(4,52)_ = 13.7, *p* < 0.001, **Figure 4C**). There were no training, group, or interaction effects for IL-6, IL-10, and TNF-α (*p* > 0.05). There was an interaction between training session and time for the IL-6/IL-10 ratio (*F*_(4,44)_ = 2.8, *p* = 0.04, **Figure 4D**), with values remaining unaltered from rest to 48 h after SIT in the first training session, but decreasing 24 and 48 h after SIT in the last training session. The IL-6/IL-10 ratio at 24 and 48 h after SIT in the last training session was also lower than at all time points of the first training session, with no difference between the PLA and CAF groups.

### Aerobic Fitness and Body Composition Post-training

Two weeks of SIT increased 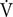
O_2max_ by ∼7% (main effect of training, *F*_(1,18)_ = 5.9, *p* < 0.05, **Table 1**) and Ẇ_max_ by ∼6% (main effect of training, *F*_(1,18)_ = 9.9, *p* < 0.05, **Table 1**), in both the CAF and PLA groups. There was no training or group effect for body mass, GET, RCP, and HR_max_ (all *F*_(1,18)_ < 3.6, *p* > 0.05, **Table 1**).

## Discussion

In this study, we demonstrated, for the first time, that the IL-6/IL-10 ratio (a marker of inflammatory status) was reduced 24 and 48 h post the last SIT session after 2 weeks of SIT. This result indicates that short-term SIT switches the late post-exercise cytokine response to an anti-inflammatory profile. We have also demonstrated that both acute caffeine intake and chronic SIT reduced the appearance of markers of exercise-induced muscle damage. However, in contrast to our initial hypothesis, caffeine ingestion prior to training did not induce a larger improvement in overall systemic anti-inflammatory status when compared to SIT performed without prior caffeine ingestion.

Consistent with previous studies, plasma IL-6 increased immediately after exercise in the present study, reaching its peak 45 min post-exercise (Zwetsloot et al., 2014). It has been suggested that IL-6, when measured immediately after the exercise, does not only emerge from inflammatory processes but can also be directly produced by the exercising muscle and can be considered a marker of muscle metabolism (Starkie et al., 2003; Petersen and Pedersen, 2005; Carey et al., 2006; Pedersen and Febbraio, 2008; Pedersen, 2012). For example, IL-6 released from skeletal muscle is increased when muscle glycogen levels are depleted, providing a signal to mobilize extra-muscular substrates (e.g., free fatty acids) (Pedersen et al., 2001; Carey et al., 2006; Pedersen and Febbraio, 2008). In addition, the acute increase of IL-6 to a given workload is less pronounced after 6 weeks of high-intensity interval training (Croft et al., 2009). In the present study, although there appeared to be training-induced adaptations in both anaerobic and aerobic metabolism, as supported by a higher plasma lactate accumulation after the last SIT session and a higher 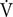
O_2max_ after training, respectively, there was no change in the exercise-induced increase in IL-6 when compared with the first and last SIT session. However, the SIT intensity was 6% larger in the last compared to the first training session, which indicates the metabolic demand was also increased. This is in accordance with previous findings showing that the exercise-induced increase of plasma IL-6 was not altered with endurance training when measured at the same relative workloads (Fischer et al., 2004), and reinforces the metabolic nature of IL-6 when measured acutely. However, it is also important to highlight that IL-6 is not only a pro-inflammatory marker but can also be an anti-inflammatory marker when measured acutely (Starkie et al., 2003; Petersen and Pedersen, 2005). In this context, IL-6 produced during exercise will induce an increase in IL-10 (Starkie et al., 2003; Steensberg et al., 2003; Pedersen, 2012). In the present study, we observed a transient increase in IL-10, peaking in the plasma 45 min after the SIT session. The IL-10 also acts to restore basal inflammatory homeostasis by inhibiting the production of pro-inflammatory TNF-α from immune cells (Starkie et al., 2003; Steensberg et al., 2003). In accordance with this, our finding showed that plasma TNF-α increased immediately after a single session of SIT, but was not significantly different from basal levels 45 min after exercise, when both IL-6 and IL-10 reached their peak in the plasma. Thus, the early time-course of measured cytokines in the present study suggests that a single session of SIT induces an IL-6 response compatible with its metabolic and anti-inflammatory actions.

An interesting finding of the present study is that following 2 weeks of SIT there was a reduction in the late (24 and 48 h after exercise) IL-6/IL-10 ratio. To the best of our knowledge, this is the first study showing that adaptation to a short-term period of SIT leads to a more anti-inflammatory profile post-exercise. Only two studies have investigated the effect of a brief training period of SIT (4–7 × 30-s sprint/240-s rest) on the cytokine response, and these studies have reported either a late increase in pro-inflammatory cytokines (Richardson et al., 2016) or no alterations (Hovanloo et al., 2013). In contrast to these studies (Hovanloo et al., 2013; Richardson et al., 2016), we applied a modified SIT protocol (more sprints and less time for recovery) with the objective of maintaining participants as close to 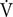
O_2max_ as possible during training. It has been demonstrated that a longer time at 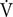
O_2max_ provides a stronger stimulus to improve the exercise-induced adaptations in aerobic fitness (Ronnestad et al., 2015). Our results suggest that a longer time expended at 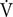
O_2max_ during SIT might also be an optimal stimulus to improve cytokine status in physically-active men. In fact, an increased late pro-inflammatory response would result in immune suppression and chronic fatigue (Smith, 2000; Bessa et al., 2016). On the other hand, a late reduction in the IL-6/IL-10 ratio indicates a positive training adaptation of the immune system (Smith, 2000; Bessa et al., 2016). In addition, we also observed a lack of increase in CK levels during the last training session in both groups. This lack of change in CK concentration might be associated with a lower muscle proteolysis that occurs with regular training (da Costa Santos et al., 2011; He et al., 2015). Therefore, performing a SIT protocol with more sprints and shorter recovery, as used in the present study, was efficient to promote a short-term improvement in the IL-6/IL-10 ratio and a decrease in markers of exercise-induced muscle damage. Furthermore, an improved balance between the release of pro- and anti-inflammatory cytokines has been proposed to benefit individuals with low-grade inflammation, such as those with chronic metabolic and cardiorespiratory diseases (Gleeson et al., 2011). This raises the possibility of using this modified SIT protocol as an intervention for the prevention and/or treatment of inflammatory diseases.

Another novel experimental approach included in the present study was that a group of individuals performed SIT sessions after they had ingested caffeine. This is the first study combining caffeine and short-term SIT. The rationale for this approach was that the cytokine response may have been altered by caffeine (Tauler et al., 2013, 2016; Rossi et al., 2017). However, the IL-6, IL-10, and TNF-α response to a single session of SIT were similar between the PLA and CAF groups in both the first and last training sessions. Although previous studies have demonstrated that caffeine intake increased the acute IL-6 and IL-10 response after a 15-km running time trial (Tauler et al., 2013, 2016), the greater cytokine release with caffeine was associated with a greater power and larger metabolism stress as indicated by a greater increase in both plasma adrenaline and lactate after exercise (Tauler et al., 2013, 2016). Therefore, caffeine may not have a direct effect on IL-6 and IL-10, but instead it is the increased power and consequently higher metabolic demand that is ultimately responsible for increasing the IL-6 and IL-10 response. This interpretation is compatible with the metabolic action of IL-6 when measured acutely (Steensberg et al., 2002; Pedersen, 2012). However, caffeine intake prevented the CK increase during the first training session, which indicates that caffeine may have a protective effect on exercise-induced muscle damage, as previously reported (da Costa Santos et al., 2011). Caffeine increases intracellular cyclic AMP (Horrigan et al., 2004), which decreases skeletal muscle protein degradation by regulating the ubiquitin–proteasome system (Goncalves et al., 2009). Therefore, caffeine seems to protect against exercise-induced muscle damage when individuals are not accustomed to performing high-intensity exercise. This effect tends to disappear as training-induced adaptations progress.

Finally, the short-term SIT program used in the present study (2 weeks, six sessions) improved 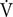
O_2max_, Ẇ_max_ and MP. This is in accordance with previous studies reporting improved aerobic and anaerobic fitness after 2 weeks of SIT (Hazell et al., 2010; Richardson et al., 2016). Therefore, performing a SIT protocol with more sprints and shorter recovery, as used in the present study, was efficient to promote improvement in aerobic fitness (∼7%) in only 2 weeks.

Some limitations of the present study must be recognized. We have investigated the effect of SIT on cytokine parameters before and after only 2 weeks of training. However, there is research indicating limited additional benefits of performing SIT for a longer period (Burgomaster et al., 2008; Richardson et al., 2016). For example, there is a report that failed to demonstrate any further significant increase in 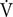
O_2max_ when SIT is performed for longer than 3 weeks (Burgomaster et al., 2008). Further, because plasma caffeine levels before each training session were not measured in the present study, we were unable to confirm caffeine abstinence via analysis of blood caffeine concentration. However, an analysis of the food records confirmed that participants did not ingest any caffeine before the tests and training sessions. In addition, we have administrated a similar dose of caffeine to previous studies (5 mg kg^-1^), which is sufficient to raise plasma caffeine to ∼36 μmol L^-1^, a concentration able to cause physiological alterations and improvement in exercise performance (Graham and Spriet, 1995). Because participants habitually ingested a small quantity of caffeine (∼100 mg day^-1^), we chose to avoid a complete caffeine withdrawal as this may have influenced adaptations to training. Therefore, participants in both groups were allowed to drink a cup of coffee (45 mg of caffeine) at breakfast on the days in which they did not train, except after training sessions 1 and 6. It seems unlikely this small amount of caffeine would have affected training adaptations, but further studies with full caffeine abstention should be performed. In addition, some participants in the CAF group were classified as non-responders, which suggests a possible individual effect of caffeine. Finally, diet was strictly controlled but we were unable to quantify the ingestion of some specific amino acids in the diet (e.g., creatine and taurine), which may have influenced exercise performance. However, the protein intake and sources of protein (mainly meat and milk) were very similar between the groups and consumption was stable during the entire study. In addition, none of the participants used any supplement of creatine or taurine before the study, and they were asked to not consume any of these supplements throughout the study. Therefore, it seems improbable that amino acids ingestion influenced our results.

## Conclusion

Two weeks of SIT improves the late exercise-induced anti-inflammatory response and reduces the exercise-induced increase in markers of muscle damage. Although caffeine ingestion did not enhance the ability of the SIT to improve physical fitness, caffeine seems to prevent exercise-induced muscle damage when individuals are not accustomed to high-intensity exercise.

## Author Contributions

GF conception, study design, data acquisition, analysis and interpretation, and wrote the manuscript. LF data acquisition and revising of the manuscript. RB study design and revising of the manuscript. DB data interpretation and revising of the manuscript. EB data analysis and revising of the manuscript. FD-O conception, study design, and revising of the manuscript. AL-S conception, study design, data analysis and interpretation, aided in the drafting and revising of the manuscript. All authors have read and given final approval of this version of the manuscript for publication.

## Conflict of Interest Statement

The authors declare that the research was conducted in the absence of any commercial or financial relationships that could be construed as a potential conflict of interest.
